# Case Report: Novel Splicing Variant in *SH2D1A* in a Patient With X-Linked Lymphoproliferative Syndrome Type 1

**DOI:** 10.3389/fped.2022.812590

**Published:** 2022-03-23

**Authors:** Won Kyung Kwon, Jee Ah Kim, Jong-Ho Park, Doo Ri Kim, Su Eun Park, Yae Jean Kim, Keon Hee Yoo, Ja-Hyun Jang, Eun Suk Kang

**Affiliations:** ^1^Department of Laboratory Medicine and Genetics, Samsung Medical Center, Sungkyunkwan University School of Medicine, Seoul, South Korea; ^2^Clinical Genomics Center, Samsung Medical Center, Seoul, South Korea; ^3^Department of Pediatrics, Samsung Medical Center, Sungkyunkwan University School of Medicine, Seoul, South Korea; ^4^Department of Pediatrics, School of Medicine, Pusan National University, Children's Hospital, Yangsan, South Korea

**Keywords:** XLP1, *SH2D1A* gene, mRNA studies, primary immunodeficiency, rare disease (RD)

## Abstract

X-linked lymphoproliferative disease type 1 (XLP1), an X-linked recessive genetic disorder, is associated with primary immunodeficiency. Patients with XLP1 are susceptible to Epstein–Barr virus (EBV) infection. *SH2D1A* gene is known as the causative gene. We found a novel hemizygous variant of *SH2D1A*, c.162_201+31delinsTACAAGGACATATACA, from a 5-year-old male patient who had been diagnosed with EBV infection and Hodgkin's lymphoma. In targeted next-generation sequencing (NGS), complex variants at exon 2 were not consistently identified with two software programs. They showed a soft-clipped read pattern. The variant had a 71-bp deletion and a 16-bp insertion across exon 2 as confirmed by direct sequencing. As the variant was located within the exon–intron boundary, two aberrant transcripts were shown by RNA study. Although NGS method has a limitation in detecting large deletion/duplication variants, proper bioinformatics pipeline and careful review of data might enable the detection of complex variants.

## Introduction

X-linked lymphoproliferative disease type 1 (XLP1) is an inherited immunodeficiency characterized by susceptibility to Epstein–Barr virus (EBV) infection (1). *SH2D1A* gene, located on chromosome Xq25, is known as the causative gene. *SH2D1A* encodes the cytoplasmic protein SAP (signaling lymphocyte activation molecule-associated protein), which has an important role in regulating T cells, NK cells, NKT cells (2, 3), and possibly B cells (4). The incidence of XLP1 in the United States was reported to be one in one million male individuals (5). About 120 different variants of *SH2D1A* were found in Human Gene Mutation Database (HGMD). Typical manifestations of XLP1 include EBV-associated hemophagocytic lymphohistiocytosis (HLH) and lymphomas (6). Up to 35% of patients have no evidence of previous EBV infection (7, 8). It has been reported that dysgammaglobulinemia (51.8 vs. 37.2%) and lymphoma (25.0 vs. 19.6%) have higher rates in EBV-positive XLP1 patients than in EBV-negative patients (8). On the other hand, EBV-negative XLP1 patients experience progression to HLH more frequently (51.0 vs. 21.4%) (8). Therefore, XLP1 is an immune dysregulation disorder not specifically associated with EBV infection (9). Due to various clinical manifestations, the clinical diagnosis for XLP1 is often difficult. Molecular approaches are helpful for its confirmatory diagnosis. Here we report a 5-year-old male patient with XLP1 caused by a novel pathogenic variant, c.162_201+31delinsTACAAGGACATATACA, in *SH2D1A*. This complex variant was suspected to have a 71-bp deletion and a 16-bp insertion based on next-generation sequencing (NGS). Such deletion and insertion were eventually confirmed by direct sequencing. It was found that the variant affected the splicing and yielded two aberrant transcripts by RNA study.

## Case Description

### Clinical Manifestations and Laboratory Findings

**Figure 1:**
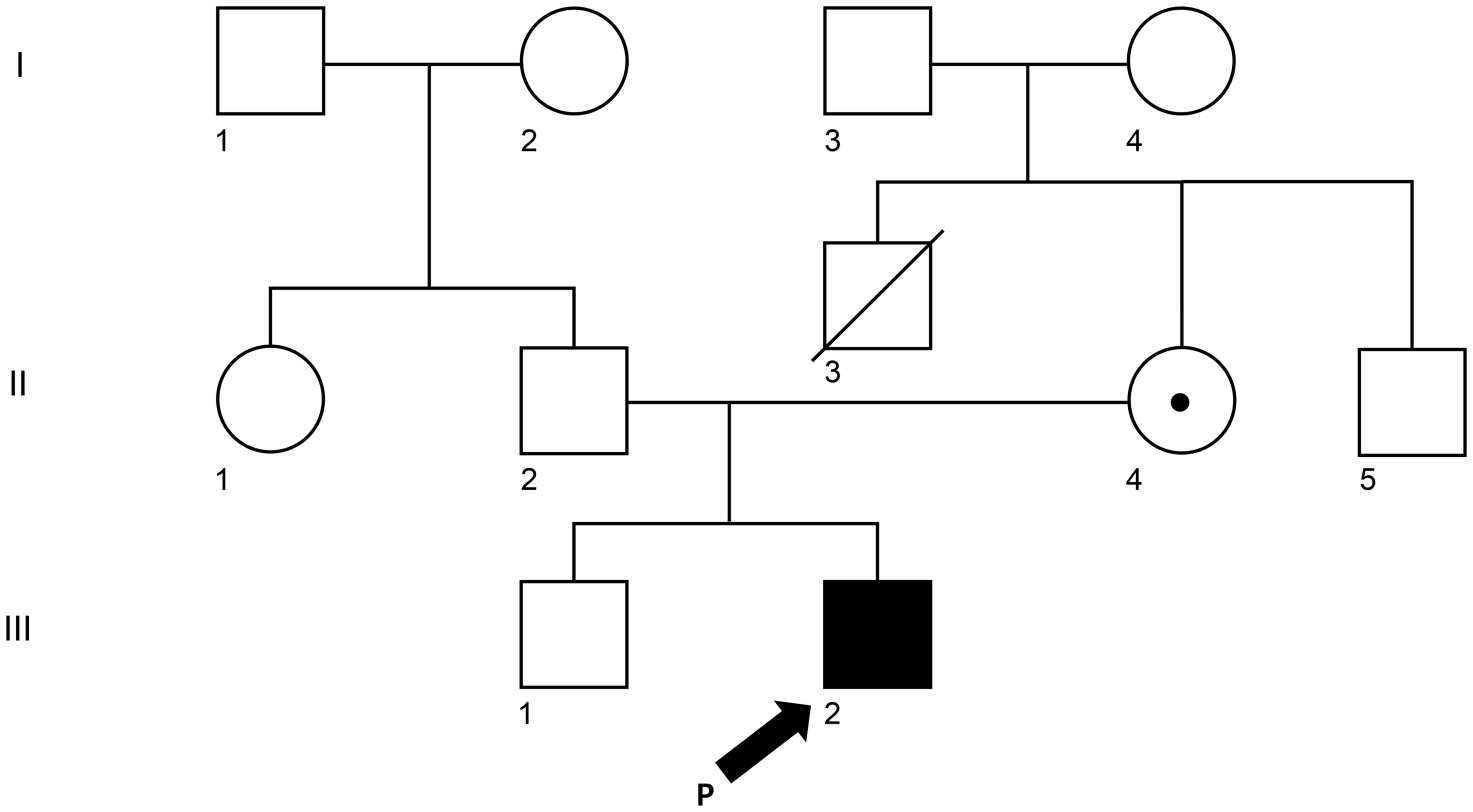
Pedigree of the XLP1 case child. The arrow (III-2) is the proband. II-3 (his mother) and III-1 (his brother) were tested for the same variant. The white square represents a male individual who was unaffected, the white circle was for an unaffected female individual, and the dot represents the mother who is a carrier.

A 5-year-old Korean male patient who had recurrent fever (up to 39°C) with high EBV titer was transferred from an outside hospital for further evaluation of primary immunodeficiency (PID). At 3 years old, he had been hospitalized for a total of three times due to multiple episodes of mycoplasma pneumonia. At that time, his immunoglobulin G was nearly absent (10.4 mg/dl, reference range: 480–900 mg/dL for 3–5 years old). However, he had normal B cell counts (1,082 × 10^9^/L, reference range: 200–2,100 × 10^9^/L). His NK cell count (67 × 10^9^/L, reference range: 100–1,000 × 10^9^/L) was decreased, although his T cell count was normal. With a presumptive diagnosis of common variable immunodeficiency, Intravenous immunoglobulin (IVIG) treatment was started. At that time, he had visited the previous hospital due to recurrent fever and diagnosis of pneumonia and bronchiectasis. He was treated with cefotaxime for 3 days. His C-reactive protein (CRP) decreased from 5.31 to 2.2 mg/dl. He was found to have an EBV titer of 35,100 copies/ml. Regarding his family history, his uncle (older brother of his mother) died from meningitis at 3 years old (Figure 1). When admitted, his body temperature was 39.0°C, and his pulse rate was 124/min. However, his breathing sound was normal. Palpable lymph node enlargement and organomegaly (except swelling in both parotid gland sites) were not observed. He had mild leukocytosis (19,450 × 10^9^/L) with neutrophilia (13,830 × 10^9^/L) and monocytosis (1,670 × 10^9^/L). He showed a markedly increased CRP level (14.16 mg/dl). Due to continuous administration of IVIG before he was transferred, his immunoglobulin levels were within the normal range. Tests for EBV were performed, including serologic tests of EBV and quantitation of EBV. The serologic tests for anti-VCA, IgG, and anti-EBNA antibodies were positive. The EBV DNA titer measured with a Real-Q EBV Quantification Kit (BioSewoom, Seoul, Korea) was 530 IU/ml. Immunophenotyping of lymphocyte subsets and naïve/memory B and T cell subsets was performed. The results are shown in Table 1. His CD16+56+3- NK cell fraction was markedly decreased both in proportion and count (1.0%, 33 × 10^9^/L). Although the CD19 + B cell proportion and count were within the reference ranges as before, both switched/non-switched (CD10-CD27+IgD+/-) and differentiated (IgD-CD27-) memory B cells were rarely detected (2.3%, 30 × 10^9^/L) with increased IgD + CD27- naïve B cell fraction. T cells also showed a skewed distribution of CD45RA+CCR7+ naïve T cells (73.2%, 441 × 10^9^/L) with decreased CD45RO + memory T cells. Radiologic studies revealed low-density lesions in the thymus, spleen, liver, and T12 level of spine bone. Multiple cervical and mediastinal lymph node enlargement with swollen left parotid gland which needed to be distinguished between inflammatory sialadenitis and EBV-related lymphoma were observed (Table 1). The pathology of lymph nodes based on video-assisted thoracoscopic surgery revealed classic Hodgkin's lymphoma, nodular sclerosis type with positive EBV by *in situ* hybridization. With a diagnosis of Hodgkin's lymphoma, a combination chemotherapy of COPP-ABV including cyclophosphamide, vincristine sulfate (Oncovin), prednisone, procarbazine hydrochloride, doxorubicin hydrochloride (Adriamycin), bleomycin, and vinblastine sulfate was started at admission day 16. After five cycles of chemotherapy, the patient underwent thoracoscopic lobectomy to remove persistent pulmonary nodules suspected to be a fungal infection. The pathology findings were consistent with inflammatory nodules composed of histiocyte aggregates and some lymphocytes. He developed a headache and left side motor weakness. The brain MRI showed multifocal brain lesions suspicious of lymphoproliferative disorder or encephalitis associated with EBV infection. After four cycles of rituximab, the abnormal hypermetabolic lesions due to EBV-related LPD resolved, with EBV DNA undetectable in the cerebrospinal fluid. Currently, the patient is awaiting haploidentical peripheral blood stem cell transplantation from his father.

**Table 1:** Clinical characteristics and laboratory, radiologic, and pathologic findings of the patient at initial diagnosis.

**Test**			**Results**
Lab findings	CBC	Hb (g/dl)/WBC (×103/μl)/Plt (×103/μl)	11.4/19,450/349 13,830/3,950/1,670
		ANC/ALC/monocyte (×103/μl)
	Chemistry	AST/ALT/ALP (IU/L) CRP (mg/dl)	29/19/176 14.16
	Immunoglobulin	IgG/IgA/IgM (mg/dl)	1,179/5/5
Lymphocyte subset	Lymphocyte subset (%)	T/B/NK (%)	55.1/42.5/1.0
	Memory B cell	Naive/memory/differentiated (%)	97.7/2.3/0.1
	Memory T cell	T4: naive/CM/EM (%) T8:	73.2/6.3/16.1 43.0/4.2/8.3/44.8
		naive/CM/EM/EMRA (%)
EBV serologic findings	EB-VCA, IgG	Positive	
	EB-VCA, IgM	Negative	
	EBV-EA	Negative	
	EBNA, IgG	Positive	
Pathologic findings	Mediastinal lymph node	Classic Hodgkin lymphoma	
		nodular sclerosis type
Radiologic findings	Neck CT	Swollen left parotid gland and multiple cervical/mediastinal lymphadenopathy. EB virus-related	
		lymphoma and lymphoproliferative disease needs to be excluded.	
	Chest CT	Multifocal low-density lesions in the thymus, spleen, and multiple enlarged lymph nodes in the	
		mediastinum which suggest lymphoproliferative disease.	
	Abdomen CT	Multiple small or enlarged lymph nodes in the mediastinum and abdomen with borderline	
		hepatosplenomegaly.	

*The lymphocyte subsets were defined as naive memory B cells—IgD+CD27-, memory B cells—CD10-CD27+IgD+/-, differentiated B cells—IgD-CD27-, naive memory T cells—CD45RA+CCR7+, central memory T cells—CD45RO+CCR7+, effector memory T cells—CD45RO+CCR7-, and EMRA—CD45RA+CCR7-. CM, central memory T cells; CT, computed tomography; EM, effector memory T cells; EMRA, CD45RA-expressing effector memory T8 cells; EB-VCA, Epstein–Barr virus capsid antigen; EBV-EA, EBV early antigen; Hb, hemoglobin; Plt, platelet count*.

### Genetic Diagnosis

NGS for targeted panel of B cell and humoral immune deficiency, including 74 related genes including *SH2D1A*, was performed. Target enrichment with customized probes (IDT, Coralville, IA, USA) and subsequent massively parallel sequencing with NextSeq550 (Illumina, San Diego, CA, USA) were performed. The sequence reads were aligned to the reference genome, hg19, with decoy sequence using the BWA-mem algorithm implemented in BWA 0.7.17 (10). Duplicate reads were marked using Picard 2.19 (https://broadinstitute.github.io/picard/). Recalibration of base quality scores was performed using previously known sites (from dbSNP138, Mills and 1000G gold standard INDELs b37 sites, and 1000G phase1 INDELs b37) after removing duplicate reads using GenomeAnalysisTK-4.1.2.0 (GATK) (10). Variants were called using HaplotypeCaller in GATK, VarDict, and Strelka2 and annotated with ANNOVAR. All variants were interpreted based on the 2015 American College of Medical Genetics (ACMG) and Genomics and the Association for Molecular Pathology (AMP) guideline (11) and, additionally, the ClinGen Sequence Variant Interpretation Workgroup's recommendation (12).

**Figure 2:**
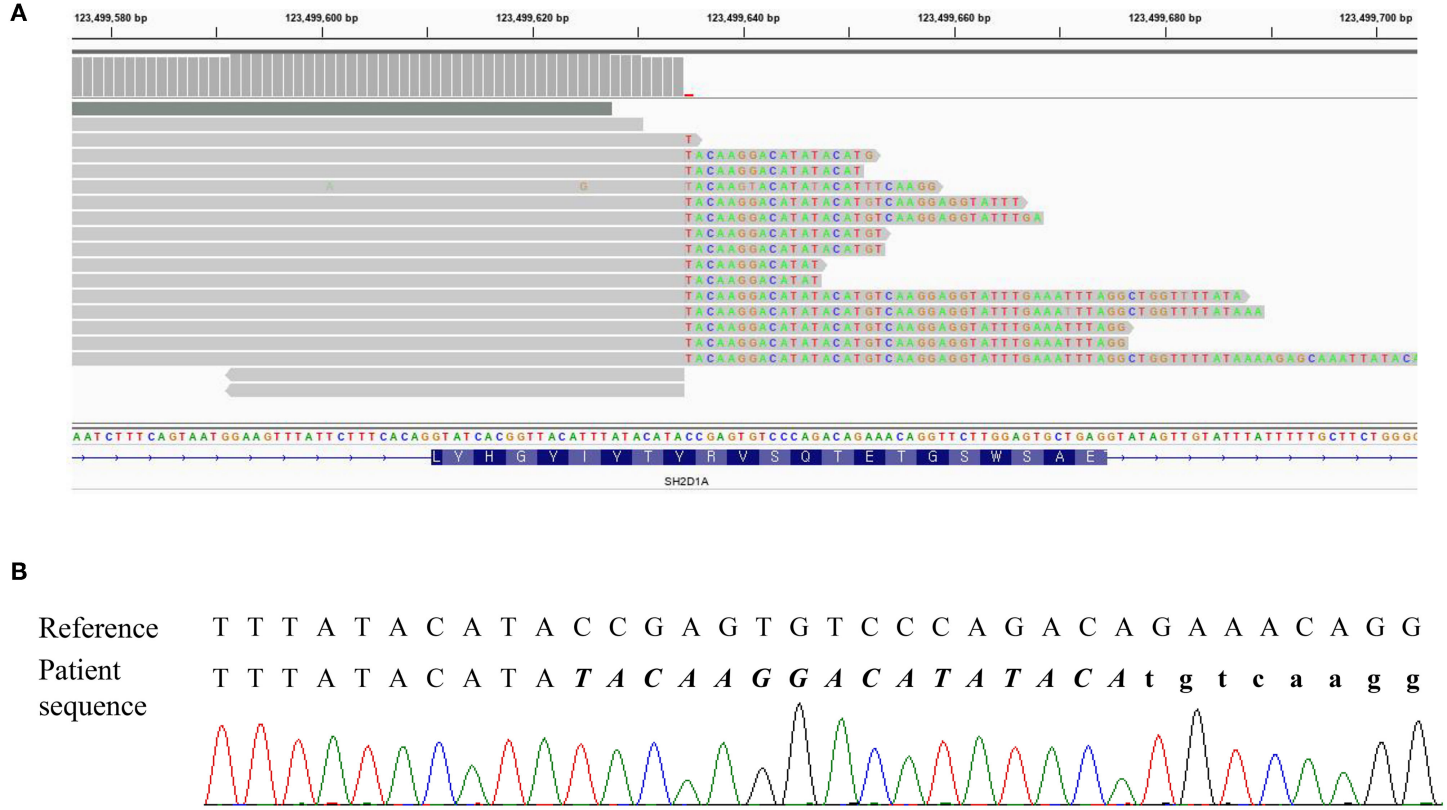
DNA result of the patient. **(A)** IGV snapshot of a next-generation sequencing panel showing soft clipped reads. **(B)** Sequencing chromatogram showing a hemizygous variant of c.162_201+31delinsTACAAGGACATATACA. The inserted sequences were written in italic letters, and the intron sequences were written in small letters.

**Figure 3:**
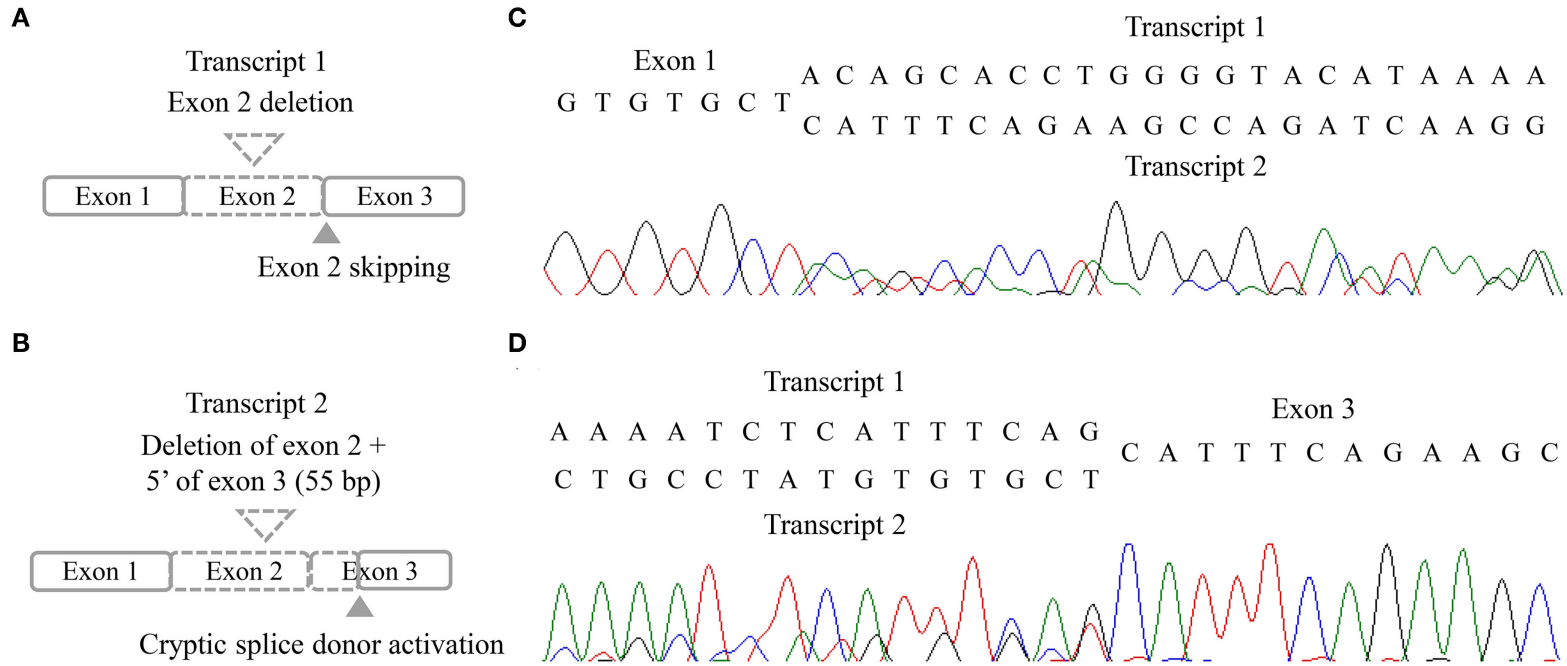
RNA results of the patient. **(A,B)** Schematic illustration of two aberrant transcripts. **(C,D)** Sequencing chromatogram in forward direction **(C)** and reverse direction **(D)**.

Three missense variants in *COPA, NLRC4*, and *DOCK8* genes, deletion/duplication, and missense variants in *SH2D1A* were identified. According to the 2015 ACMG-AMP guideline, the missense variants in *COPA, NLRC4*, and *DOCK8* genes were classified as variants of uncertain significance. Variants of the *COPA* gene (NM_004371.3:c.3502A > G) had one moderate and support pathogenic evidences [low allele frequency (0.00119%) and a low rate of benign missense variant in this gene] but one support benign evidence [*in silico* analysis (REVEL 0.1)]. The variant of the *NLRC4* gene showed no evidence. Only a heterozygous variant was detected in the *DOCK8* gene with an autosomal recessive inheritance pattern and a low allele frequency (0.00080%). For *SH2D1A*, several variants including deletion/duplication and missense changes located at exon 2 were called. Called by only GATK with low depths (5-11X) but not with other callers (Vardict and Strelka2), it was assumed as a false positive calling at first. A manual review of BAM file using the Integrated Genome Browser was performed to exclude whether exon deletion was present. Visual inspection revealed soft-clipped reads which suggested a large deletion/insertion event at the 3′ of exon 2, including the flanking intron (Figure 2A). Targeted direct sequencing revealed that the variant had a deletion of 71 bp and an insertion of 16 bp across exon 2 with a flanking intron [NM_002351.4 (SH2D1A):c.162_201+31delinsTACAAGGACATATACA] (Figure 2B). Since deletion/insertion involved the splice donor site, RNA study using peripheral blood leukocytes was performed to investigate the effect of such deletion/insertion on splicing. Reverse transcription-PCR and complementary DNA sequencing were performed across exons 1–3 of *SH2D1A* using primers designed by the authors. Gel electrophoresis showed two aberrant mRNA transcripts. One transcript corresponded to exon 2 skipping (r.138_201del, p.Tyr47_Glu67del). The other lacked exon 2 and 55 bp of exon 3 by creating a new splice acceptor site (r.138_256del, p.Tyr47Ilefs^*^17) (Figure 3). Finally, the variant could be classified as likely a pathogenic variant (PVS1, PM2). By targeted direct sequencing, it was confirmed that his mother was a heterozygous carrier, while his unaffected brother did not have this variant.

## Discussion

XLP1 is a rare disease caused by *SH2D1A* gene which encodes for SAP protein with 128 amino acids in length (2). XLP1 patients are known to be associated with fatal infectious mononucleosis, lymphoma, and dysgammaglobulinemia (13). We report a case of a 5-year-old male patient diagnosed as XLP1 based on clinical manifestation and genetic test. He presented with multiple episodes of infection, markedly reduced IgG level, multiple lymph node enlargements, and lymphoproliferative lesions with elevated EBV DNA titer comparable with typical XLP1. The patient was found to have a disease-causing variant in *SH2D1A*. However, the variant was not accurately called by the NGS method initially due to “soft clipping” which masked reads that did not align. We suspected abnormality in that region by visual inspection of BAM file and identified the deletion/insertion variant, NM_002351.4:c.162_201+31delinsTACAAGGACATATACA. The RNA study revealed two aberrant transcripts. There are approximately 120 pathogenic/likely pathogenic variants of *SH2D1A* gene in HGMD, of which gross deletion/duplication variants, including 31 unique variants, account for more than 20% (14). Since the introduction of NGS method for clinical practice, the genetic diagnosis of single-gene disorders, including PID, has become more feasible, and diverse causative variants have been discovered. Considering that *SH2D1A* has high rates of large deletion/duplication variants, caution is needed for NGS data interpretation of *SH2D1A*. Although the NGS method has a limitation in detecting large structural variants, review of coverage, especially for X-linked disorders for male patients, use of multiple variant callers, and visual inspection of BAM file could raise the diagnostic yield. In addition, in case of including splicing sites, more detailed studies, such as mRNA studies, are needed.

## Data Availability Statement

The original contributions presented in the study are included in the article/supplementary material, further inquiries can be directed to the corresponding author/s.

## Ethics Statement

The studies involving human participants were reviewed and approved by Samsung Medical Center IRB. Written informed consent to participate in this study was provided by the participants' legal guardian/next of kin. Written informed consent was obtained from the individual(s), and minor(s)' legal guardian/next of kin, for the publication of any potentially identifiable images or data included in this article.

## Author Contributions

DK, SP, YJK, and KY cared for the patient and collected the clinical data of the patient. J-HP performed the genetic test. WK, JK, J-HJ, and ESK analyzed the gene test results and drafted the manuscript. All authors read and approved the submitted version.

## Conflict of Interest

The authors declare that the research was conducted in the absence of any commercial or financial relationships that could be construed as a potential conflict of interest.

## Publisher's Note

All claims expressed in this article are solely those of the authors and do not necessarily represent those of their affiliated organizations, or those of the publisher, the editors and the reviewers. Any product that may be evaluated in this article, or claim that may be made by its manufacturer, is not guaranteed or endorsed by the publisher.
